# Haloarchaeal carotenoids exert an in vitro antiproliferative effect on human breast cancer cell lines

**DOI:** 10.1038/s41598-023-34419-x

**Published:** 2023-05-02

**Authors:** Micaela Giani, Yoel Genaro Montoyo-Pujol, Gloria Peiró, Rosa María Martínez-Espinosa

**Affiliations:** 1 Biochemistry, Molecular Biology, Edaphology, and Agricultural Chemistry Department, Faculty of Sciences, University of Alicante, Ap. 99, 03080 Alicante, Spain; 2 Applied Biochemistry Research Group, Multidisciplinary Institute for Environmental Studies “Ramón Margalef” University of Alicante, Ap. 99, 03080 Alicante, Spain; 3 Breast Cancer Research Group, Research Unit, Dr. Balmis University General Hospital, and Alicante Institute for Health and Biomedical Research (ISABIAL), Pintor Baeza 12, 03010 Alicante, Spain; 4 Department of Pathology, Dr. Balmis University General Hospital, and Alicante Institute for Health and Biomedical Research (ISABIAL), Pintor Baeza 12, 03010 Alicante, Spain; 5 Biotechnology Department, Immunology Area, Faculty of Sciences, University of Alicante, Ap. 99, 03080 Alicante, Spain

**Keywords:** Breast cancer, Biochemical assays

## Abstract

Oxidative stress has been linked to the onset and progression of different neoplasia. Antioxidants might help prevent it by modulating biochemical processes involved in cell proliferation. Here, the aim was to evaluate the in vitro cytotoxic effect of *Haloferax mediterranei* bacterioruberin-rich carotenoid extracts (BRCE) (0–100 µg/ml) in six BC cell lines, representative of the intrinsic phenotypes and a healthy mammary epithelium cell line. Cell index values were obtained using xCELLigence RTCA System. Furthermore, cell diameter, viability, and concentration were measured at 12 h, 24 h, and 30 h. We found that BC cells were selectively affected by BRCE (SI > 1, p < 0.005). After 30 h, the population of BC cells exposed to 100 µg/ml was 11.7–64.6% of the control (*p* = 0.0001–0.0009). Triple-negative cells were significantly affected [MDA-MB-231 (IC_50_ 51.8 µg/ml,* p* < 0.0001) and MDA-MB-468 (IC_50_ 63.9 µg/ml,* p* < 0.0001)]. Cell size was also reduced after 30 h treatment in 3.8 (± 0.1) µm and 3.3 (± 0.02) µm for SK-BR-3 (*p* < 0.0001) and MDA-MB-468 (*p* < 0.0001), respectively. In conclusion, *Hfx. mediterranei* BRCE exerts a cytotoxic effect on BC cell lines representative of all studied intrinsic subtypes. Furthermore, results obtained for MDA-MB-231 and MDA-MB-468 are very promising, considering the aggressive behaviour of the triple-negative BC subtype.

## Introduction

Halophilic archaea or haloarchaea are extremophilic microorganisms that require a hypersaline environment to thrive^1^. These microorganisms synthesize a rare C_50_ carotenoid called bacterioruberin (BR) and its derivatives monoanhydrobacterioruberin (MABR) and bisanhydrobacterioruberin (BABR)^2,3^. In addition, although to a lesser extent, they also synthesize astaxanthin, zeaxanthin, lycopene, and β-carotene^3–5^. *Haloferax mediterranei* is particularly relevant among haloarchaea since it has been used as a model organism for studying numerous pathways^6–9^. Furthermore, its carotenoid production can be easily enhanced by altering cell culture conditions^10–15^.

Carotenoids are well-known for their health-beneficial properties, including antioxidant, antiproliferative, antitumoral, and immunomodulatory activities^16^. Nevertheless, C_50_ carotenoid properties have not been addressed lately^12,17–21^. Haloarchaeal carotenoids have excellent antioxidant activity^12,19,21–28^, but there is still scarce information concerning their potential effects on human health. Recent research has revealed that haloarchaeal carotenoids exerted in vitro anticancer activity in a few colorectal, breast, liver, and cervical cancer cell lines^20^. However, much research needs to be done to support these preliminary results and draw a firm conclusion.

Breast cancer (BC) is still the leading cause of death (7%) in women worldwide^29^. Breast tumours can be classified into four subtypes according to the presence or absence of hormone receptors (estrogen (ER) and progesterone receptors (PR)) and the overexpression of human epidermal growth factor receptor 2 (HER2) in combination with a set of clinical features. The four subtypes were named luminal A (ER^+^ PR^+^ HER2^−^), luminal B (ER^+^ PR HER2±), HER2-enriched (ER^−^ PR^−^ HER2^+^), and triple-negative (ER^−^ PR^−^ HER2^−^) BC^30,31^. Despite the advanced BC treatments in recent years, there is still a need for bioactive compounds that can help in the prevention and/or treatment of this pathology. Hence, this in vitro work aimed to determine if a *Hfx. mediterranei* BRCE was cytotoxic to BC cell lines representative of each subtype plus a mammary epithelium cell line as a control to evaluate the cytotoxicity in healthy tissue. In addition, we investigated the effect of the BRCE treatment on total viable cell number, cell adhesion, and cell diameter. Thus, this study contributes to the knowledge of the potential therapeutic applications of haloarchaeal carotenoids in BC.

## Methods

### Culture medium, pigment extraction, and quantification

*Hfx. mediterranei* R-4 (ATCC33500) was grown in a complex medium containing 12.5% (w/v) of inorganic salts^10,11,32^, 0.5% (w/v) yeast extract (*Condalab*; Madrid, Spain) and 1.5% (w/v) d(+)-Glucose anhydrous *BioChemica* (*Panreac AppliChem*; Barcelona, Spain). The pH was buffered using 30 mM Tris (*Panreac AppliChem*; Darmstadt, Germany) and adjusted to a pH of 7.3. Growth conditions included 36.5 °C and shaking at 170 rpm in a shaking incubator (*Infors HT Multitron Standard;* provided by Proquilab, Alicante, Spain) based on the data reported by Montero-Lobato and Giani et al.^10,11^. First, cells were incubated as described elsewhere^12^. Then, cells were centrifuged at 7800 rpm for 30 min to remove the supernatant and were washed twice with a 10% (w/v) inorganic salts solution, plus the last wash with distilled water to remove all remnants of salt and induced cell lysis. Cell pellets were kept at − 20 °C until further use.

### Carotenoid extraction and BR quantification

Pure acetone of HPLC grade (*Panreac AppliChem*, Panreac Quimica, Barcelona, Spain) was added to the cell pellets in a ratio of 1 ml of acetone per 10 ml cell culture^10^. After that, a 4 °C overnight incubation and later centrifugation (7800 rpm, 30 min) were necessary to obtain the BRCE. As a result, BR concentration was calculated as follows^10^:

$${\text{mg}} \cdot {\text{L}}^{ - 1} = ({\text{OD}}_{494} /2540) \times 10^{4}$$

BRCE were stored at − 20 °C in solution.

*Hfx. mediterranei* BRCE obtained under the conditions described in section “Culture medium, pigment extraction, and quantification” contains 75.5 (± 1.9)% of BR^12^.

### Preparation of a stable, biocompatible solution

Considering that carotenoids were solubilized in acetone, we aimed to replace this organic solvent with a solution compatible with human cells so that in evaluating the anticancer activity, we could ensure that the pigments caused the effects observed. Therefore, after BR quantification, acetone was removed by evaporation with a centrifugal vacuum concentrator system (*Eppendorf Concentrator 5301; Hamburg, Germany*). Then, a mammalian cell culture media (DMEM (Dulbecco's modified Eagle's medium) F-12 (1:1) with l-glutamine and 15 mM HEPES (*Biowest;* Nuaillé, France) supplemented with 10% fetal bovine serum (FBS) (*Biowest*; Nuaillé, France), and 1% penicillin (50 U/ml) and streptomycin (50 mg/ml) (*Biowest;* Nuaillé, France) was added to the BRCE up to a final concentration of 150 µg/ml. Sonication with an ultrasonic probe (*Branson SFX 550*) (*Emerson;* Dietzenbach, Germany) was required to ensure total solubilization. Previous assays were carried out to optimize sonication time. Therefore, both the solubilization and stability of the pigments were guaranteed. The samples were kept on ice, and the sonication conditions with microtip were pulsed on-time of 20 s, off-time of 40 s, amplitude of 20%, and a total duration of 4 min. The sonication cycle was repeated with off-times of 5 min between cycles to ensure the cooling of the sample. Filtration using 0.2 µm filters was carried out to guarantee sterilization of the solution before cell treatment.

### Cell lines and cell culture conditions

One healthy epithelial mammary cell line (184A1) and six human BC cell lines representative of each intrinsic subtype were used in this study (all of them commercial cell lines): Luminal A (MCF-7 and T-47D), Luminal B (BT-474), HER2-enriched (SK-BR-3), and triple-negative (MDA-MB-468 and MDA-MB-231). Since no analytical studies were carried out to confirm the absence of trace quantities of acetone, 184A1 was used as a control to evaluate the effect of the treatment on non-tumour cells and to normalize the results when compared with tumour cells. They were all grown in a monolayer and maintained as adherent cell cultures in DMEM F-12 with stable l-glutamine and 15 mM HEPES (*Biowest;* Nuaillé, France) medium supplemented with 10% FBS (*Biowest;* Nuaillé, France) and 1% penicillin–streptomycin (*Biowest;* Nuaillé, France). DMEM F-12 was selected as culture media for all cell lines to reduce variability and to ensure the nutrition requirements of all cell lines, considering that it is one of the most enriched available culture media^33^. All cell lines were incubated at 37 °C in a humified atmosphere with 5% CO_2_.

### Determination of the effect of cell adhesion on human mammary normal and BC cell lines using xCELLigence Real-Time Cell Analysis (RTCA)

xCELLigence real-time RTCA DP Instrument (3 × 16, Serial No. 32.1.2002-2536-6) (*Agilent Technologies, San Diego, CA*) was used to evaluate cell adhesion over time^34^. The equipment took measurements every 15 min for 100 h to ensure the achievement of the complete curve.

For this experiment, xCELLigence RTCA was set up according to the manufacturer's instructions. The instrument was placed inside a humidified CO_2_ incubator (*Binder CBS 170;* Tuttlingen, Germany) using the external computer system *RTCA software Pro 2.3.4* (*Agilent;* Madrid, Spain). First, background reading was done with DMEM F-12 (*Biowest;* Nuaillé, France) culture media. Next, cells were seeded to the 16 well microplates (E-Plate 16 PET; *Agilent;* Madrid, Spain). All steps were performed under sterile conditions. Firstly, 25,000, 50,000, and 75,000 cells were seeded to determine the seeding density for the rest of the experiments. The objective was to determine an initial cell density allowing later measurements at different stages of cell growth (early exponential, mid-exponential, and late-exponential) in 96-well plates (see section “Determination of the anticancer activity of *Hfx. mediterranei* carotenoids”). Once initial cell density was established, the effect of the treatment on cell adhesion was evaluated by adding a total volume of 200 µl culture media with 50,000 cells to each well. After 12 h, the cell culture volume was removed, and 200 µl of the treatment (10–100 µg/ml BR) was added in duplicates. Cell adhesion was monitored for 100 h in all cell lines. The addition of dimethyl sulfoxide (DMSO) (*Serva*; Heidelberg, Germany) and DMEM F-12 was used as the positive and negative control, respectively.

### Determination of the anticancer activity of *Hfx. mediterranei* carotenoids

Normal mammary epithelium and BC cells were seeded in 96-well cell culture plates with a density of 50,000 cells/well. After 12 h (to ensure complete cell attachment), culture media was removed, and serial concentrations ranging from 10 to 100 µg/ml BRCE were added. DMSO and DMEM F-12 were used as the positive and negative control, respectively. After 12 h, 24 h, and 30 h of treatment, cells grown in each plate well were harvested using trypsin–EDTA (0.05%) (*Capricorn Scientific;* Ebsdorfergrund, Germany). Total cell number, viability, and diameter were analyzed using a CASY OLS cell counter and analyzer (CASY TTT) (*OMNI Life Science,* Bremen, Germany). Cell adherence was assessed using xCELLigence Real-Time Cell Analysis System. Half maximal inhibitory concentration (IC_50_) for the total viable cells was calculated using GraphPad 7 Software (*GraphPad Software*; Dotmatics; San Diego, California, USA). In addition, the selectivity index (SI), defined as the ratio of IC_50_ for normal cells to that for BC cell lines, was calculated to evaluate the toxicity of the BRCE studied against normal cells and to predict their therapeutic potential.

### Microscopy

Cell images were acquired with an optical microscope (Nikon Eclipse TS100; *Nikon Instruments*; Amstelveen, The Netherlands) using a 10 ×/0.25 objective.

### Statistical analysis

Data are expressed as the mean ± standard deviation (SD). Statistical significance was calculated by ANOVA (followed by Dunnet's test for multiple comparisons) analysis using GraphPad 7 software (*GraphPad Software*; Dotmatics; San Diego, California, USA). The differences were considered statistically significant at *p*-values < 0.05*, < 0.005**, < 0.0005***, < 0.0001****. All experiments were repeated at least three times unless otherwise indicated.

## Results

### Haloarchaeal carotenoids affect cell adhesion of BC cell lines

In this experiment, the effect of *Hfx. mediterranei* BRCE (10–100 µg/ml) on the adhesion of human epithelial mammary and BC cell lines was studied using the xCELLigence RTCA system.

The curves generated by the cell index representation can give us information about the proliferation dynamics of each cell line^34^. For this reason, this equipment was also used to establish the optimal cell quantity to seed for the subsequent experiments.

**Figure 1:**
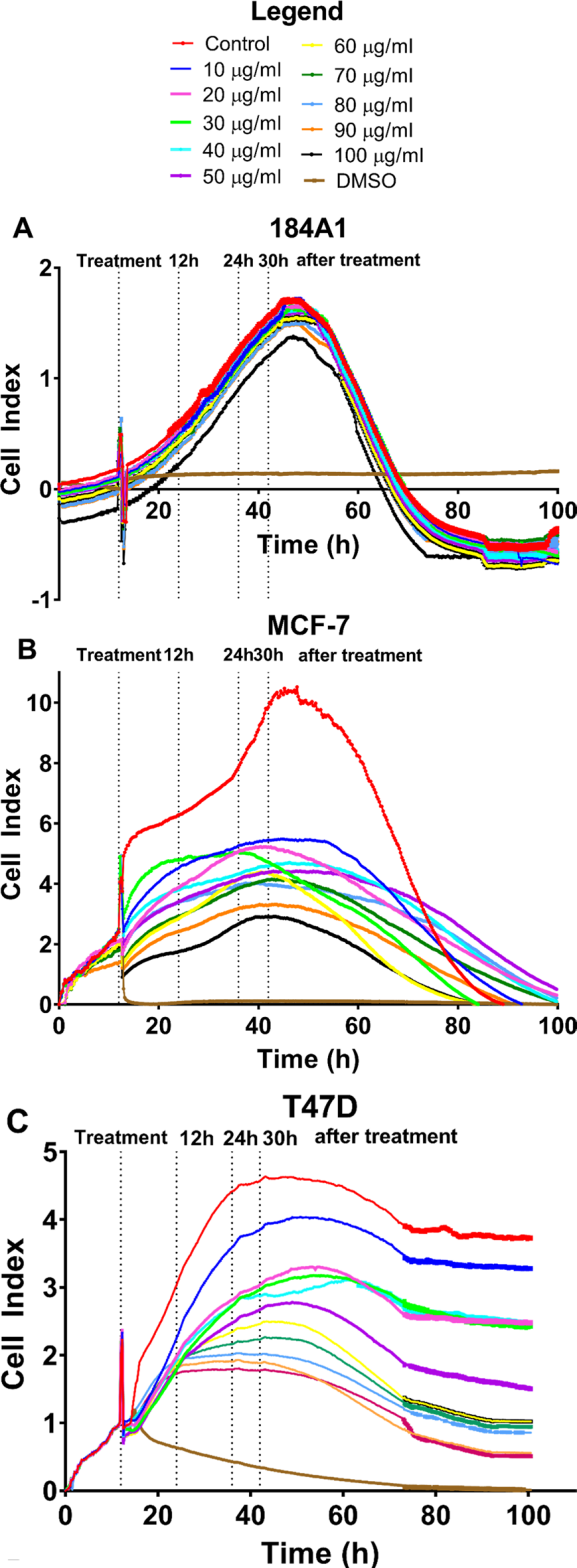

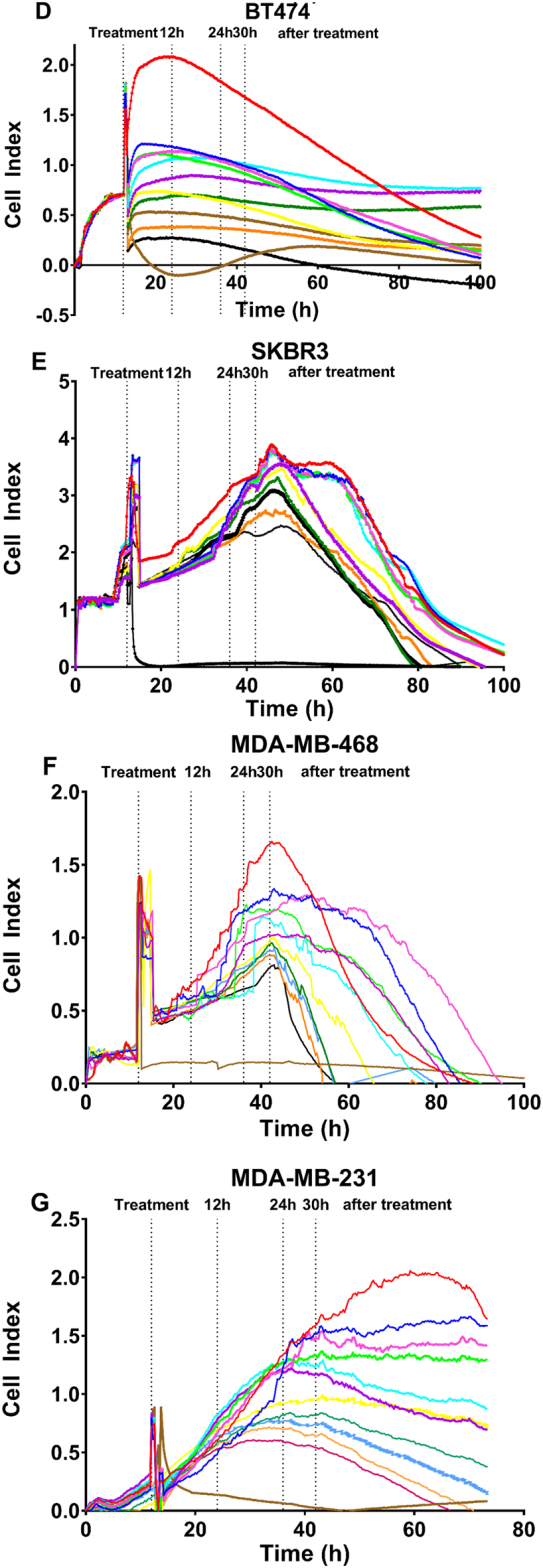
Effect of BRCE treatment on adherence represented as cell index units of the human mammary epithelial cell line (184A1) and the representative cell lines of each BC intrinsic subtype.

Figure 1 illustrates that the adherence of all BC cells was altered when exposed to increasing concentrations of the carotenoid treatment in a concentration-dependent manner (Fig. 1B–G). In contrast, the healthy mammary cell line (184A1) was less affected than tumour cell lines (Fig. 1A), which implies that the treatment exerts a selective effect.

**Figure 2:**
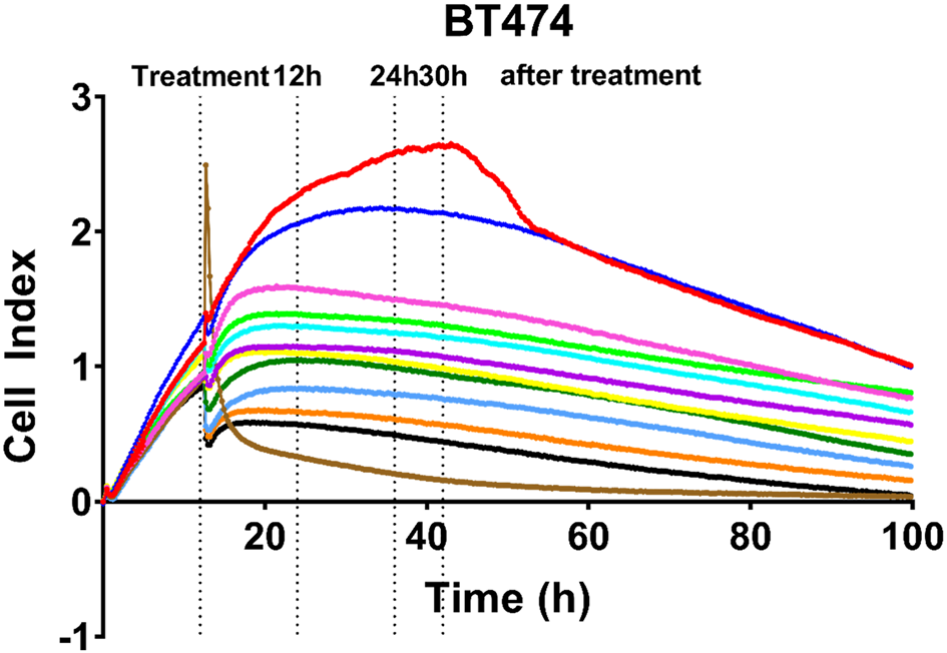
Effect of BRCE treatment on adherence represented as cell index units of the BT-474 cell line with 25,000 initial cell density.

An initial cell density of 50,000 cells was chosen to keep homogeneity because, in most cell lines, this density ensured that three measurements could be made at three stages of growth (early, mid, and late exponential phases). However, this number of cells in the BT-474 cell line was excessive for proper measurements, as shown in Fig. 1D. Therefore, although cell index decrease was detected despite high cell density, the assay was repeated by seeding 25,000 cells (Fig. 2) to analyze cell adhesion, viability, diameter, and the total number of viable cells as it was carried out with the other cell lines.

In Fig. 2, the resulting curve after 25,000 cell seeding can be observed. In this case, a concentration-dependent tendency was identified with a decrease of 90% in cell index at the highest concentration tested (100 µg/ml). Hence, it was critical to establish a growth curve that allowed us to take measurements at 12 h, 24 h, and 30 h after treatment without coinciding with the death of the control cell due to exhaustion of the nutrients present in the culture media, as it happens in Fig. 1D.

When considering the different intrinsic subtypes, the IC_50_ values at 30 h (Table 1) indicated that the most significantly affected cell lines were BT-474 (25,000 initial cell density), MCF-7, and T-47D (all *p* < 0.0001).

**Table 1:** Effect of *Hfx. mediterranei* BRCE on cell adhesion in terms of half maximal inhibitory concentration (IC_50_) (µg/ml) and selectivity index (SI) 30 h after treatment. **p < 0.005, ****p < 0.0001.

Cell line	Adhesion (Cell Index) IC_50_ (µg/ml)	Selectivity Index (µg/ml)
184A1	> 100	1
MCF-7	43.8 (± 0.06)****	2.3 (± 0.003)****
T-47D	55.1 (± 0.02)****	1.8 (± 0.001)****
BT-474 (50K)	47.6 (± 0.5)****	2.1 (± 0.02)****
BT-474 (25K)	36.9 (± 0.5)****	2.7 (± 0.04)****
SK-BR-3	93.7 (± 0.1)****	1.1 (± 0.001)**
MDA-MB-231	73.2 (± 0.2)****	1.4 (± 0.003)****
MDA-MB-468	58.23 (± 0.1)****	1.7 (± 0.008)****

A SI was estimated to determine the cytotoxicity of the compound to the normal cells (Table 1). The IC_50_ of 184A1 cells could not be calculated since 75% inhibition was obtained with the highest concentration tested (100 µg/ml). Consequently, this concentration was used as IC_50_ without a better option. Thus, the SI is an underestimation of the real potential value. An SI > 1.0 is considered a favourable value since it indicates a drug with higher efficacy against tumour cells than against normal cells^35^. *Hfx. mediterranei* carotenoids present an SI > 2 in MCF-7 and BT-474 cell lines (Table 1). A SI = 1.4–1.7 (*p* < 0.0001) was obtained for MDA-MB-231 and MDA-MB-468 cell lines representing triple-negative subtype. Furthermore, the highest concentration tested led to an approximately 60–70% reduction of cell index in these cell lines.

Of note, perturbance in all curves is observed at 12 h due to plate removal from the equipment to add treatment.

### *Hfx. mediterranei* BRCE effect on diameter, viability, and total viable cell number

#### BC cells diameter is altered in the presence of BRCE

**Figure 3:**
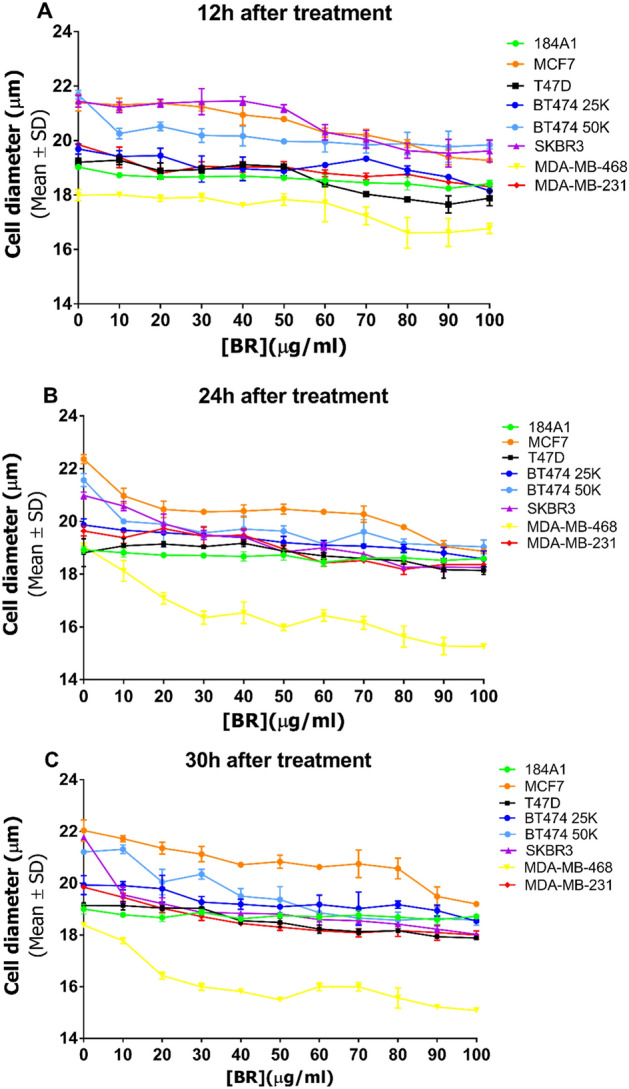
Effect of BRCE treatment on the diameter of viable cells 12 h (**A**), 24 h (**B**) and 30 h (**C**) after treatment.

Regarding human mammary epithelial cells (184A1), after 30 h treatment, their diameter did not seem to be affected by the treatment, with an average diameter of 18.9 (± 0.2) µm (Fig. 3C). As for BC cell lines, a decrease in cell size was observed, which was accentuated with time (Fig. 3A–C). After 12 h, there was a cell size difference of 2.1 (± 0.2) µm (MCF-7) (*p* < 0.0001), 1.3 (± 0.2) µm (T-47D) (*p* = 0.006), 1.5 (± 0.1) µm (BT-474 25K) (*p* = 0.0006), 1.8 (± 0.2) µm (SK-BR-3) (*p* < 0.0001), 1.2 (± 0.3) µm (MDA-MB-468) (*p* = 0.02) and 1.5 (± 0.2) µm (MDA-MB-231) (*p* = 0.0005) at the highest concentration (100 µg/ml) (Fig. 3A). After 30 h treatment, these cell size reductions were even significantly more apparent: 2.8 (± 0.4) µm (MCF-7) (*p* < 0.0001), 3.8 (± 0.1) µm (SK-BR-3) (*p* < 0.0001), 3.3 (± 0.02) µm (MDA-MB-468) (*p* < 0.0001), 1.8 (± 0.1) µm (MDA-MB-231) (*p* < 0.0001), and except for 1.3 (± 0.1) µm (T-47D) (*p* = 0.0005), and 1.4 (± 0.3) µm (BT-474 25K) (*p* = 0.0001), whose diameters remained constant.

**Figure 4:**
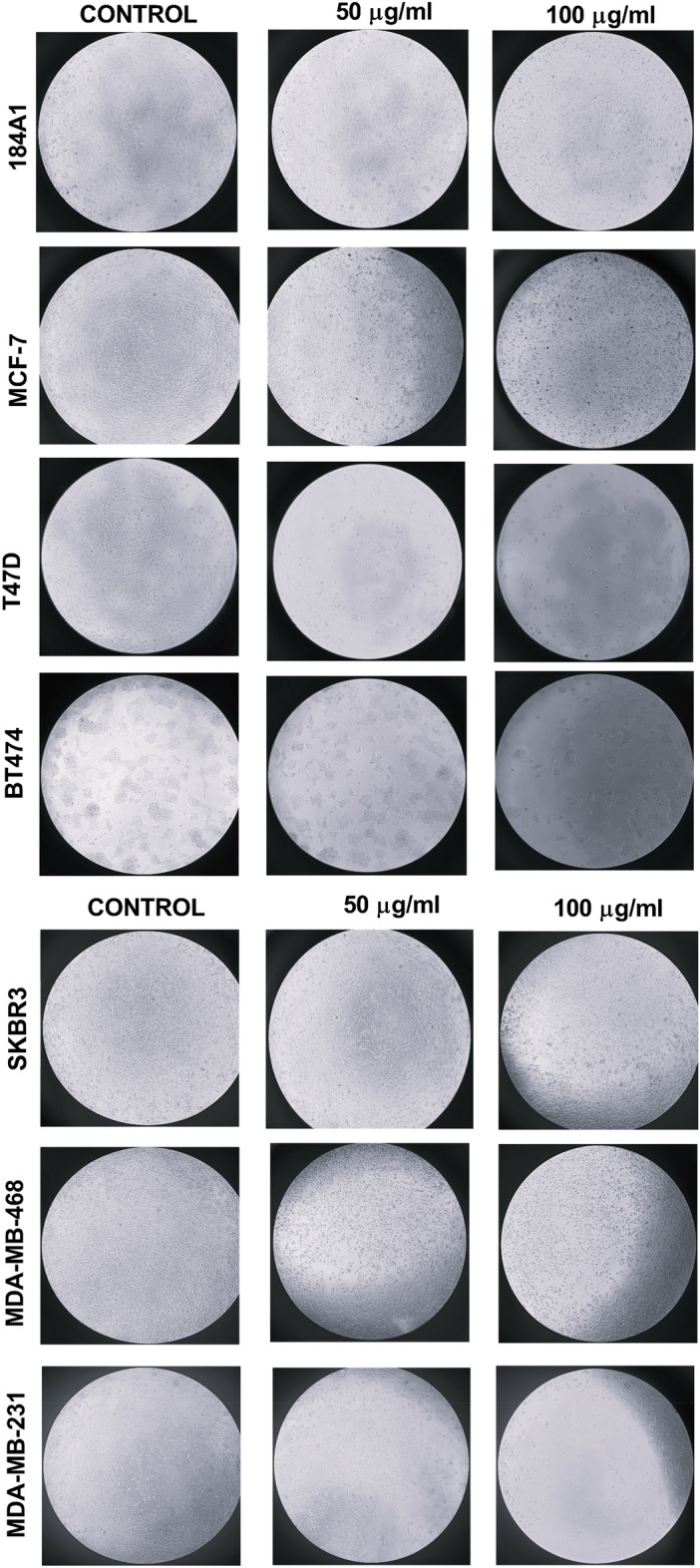
Effect of BRCE (50 µg/ml and 100 µg/ml) after 30 h treatment on cell density and morphology in normal epithelial mammary cell culture and representative cell lines of each intrinsic subtype.

Concerning Fig. 4, distinct differences in morphology were observed for MCF-7 and SK-BR-3 after 30 h of 50 µg/ml and 100 µg/ml treatment. An evident decrease in the cell population was also observed in T-47D, BT-474, MDA-MB-468, and MDA-MB-231, confirmed with population cell counting in section “Effect of *Hfx. mediterranei* BRCE on BC cell viability”.

#### Effect of *Hfx. mediterranei* BRCE on BC cell viability

**Figure 5:**
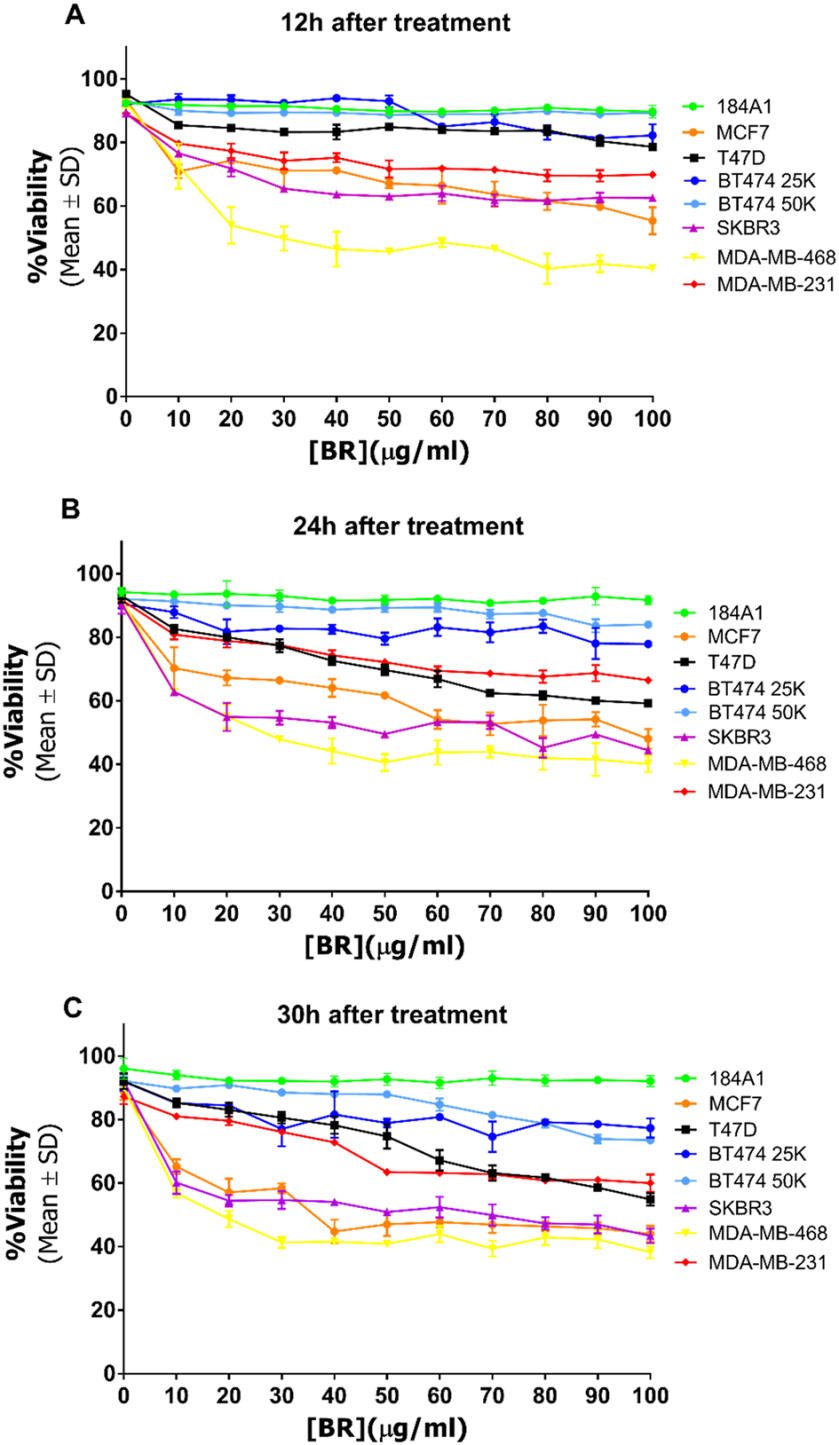
Effect of haloarchaeal carotenoid treatment on viability at 12 h (**A**), 24 h (**B**) and 30 h (**C**) after treatment.

Figure 5 shows the viability percentages after 12 h, 24 h, and 30 h of treatment. The treatment did not affect the viability of the 184A1 cell line, which agrees with the previous results presented in this study. Thus, confirming the selective effect of the extract.

After 12 h treatment, BC cell viability was already diminished at 100 µg/ml. The most affected cell lines were MDA-MB-468 (40.4 (± 0.4)%) (*p* < 0.0001) and MCF-7 (55.4 (± 4.3)%) (*p* < 0.0001), followed by SK-BR-3 (62.3 (± 1.1)%) (*p* < 0.0001) and MDA-MB-231 (69.9 (± 0.7)%) (*p* < 0.0001). After 30 h, T-47D viability dropped to 54.9 (± 2.0)% (*p* < 0.0001), joining the previously mentioned cell lines. In contrast, BT-474 (25K) viability was only reduced by 22.7 (± 3.0)% (*p* < 0.0001). Interestingly, although affected, cell viability was not reduced below 50% in most cases. In consequence, IC_50_ values were not calculated for this section.

#### Effect of *Hfx. mediterranei* BRCE on the total number of viable BC cells

**Figure 6:**
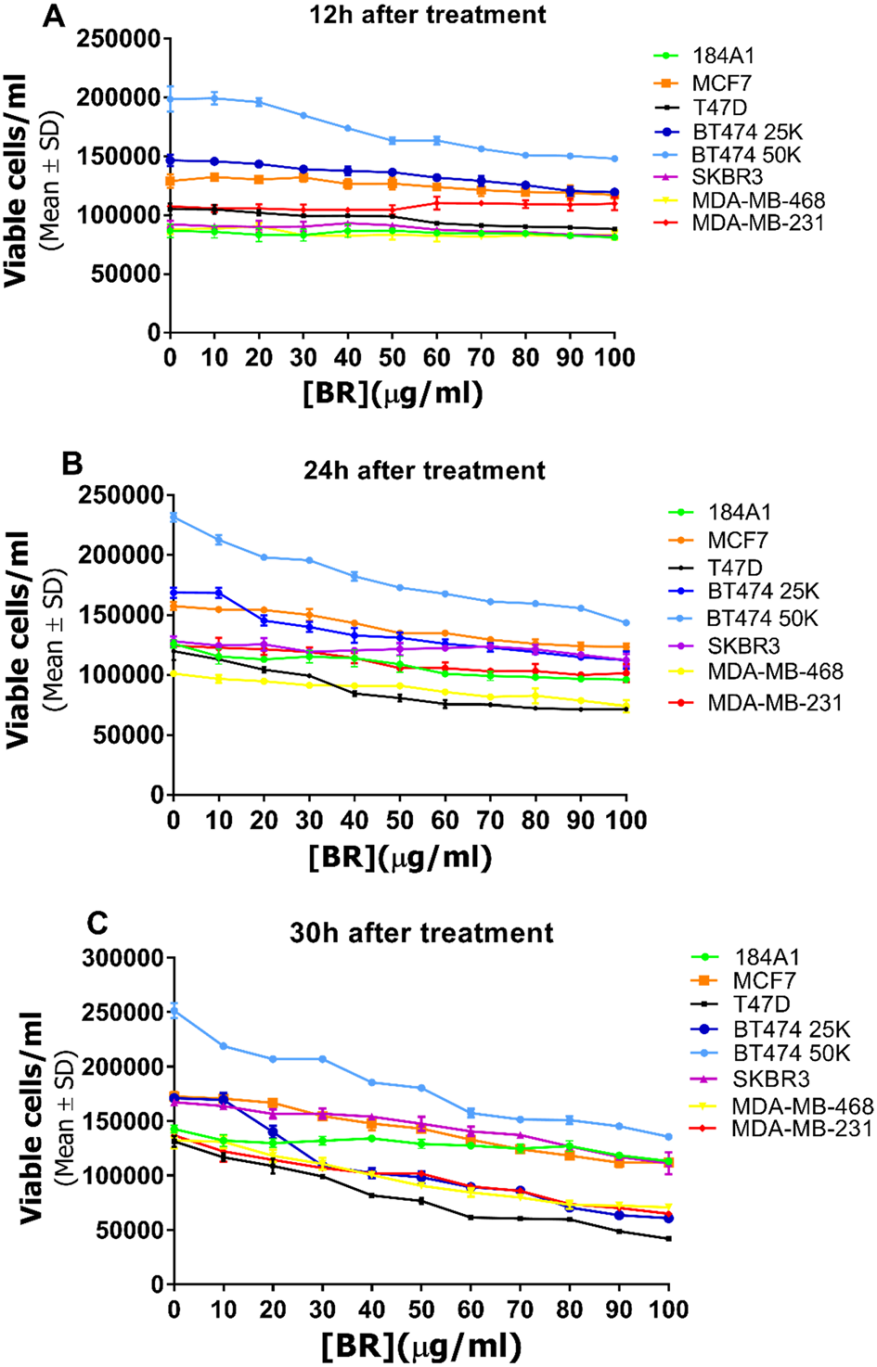
Effect of haloarchaeal BRCE treatment on concentration of viable cells at 12 h (**A**), 24 h (**B**) and 30 h (**C**) after treatment.

Considering the significant results obtained in the xCELLigence curves of the effect of the haloarchaeal BRCE treatment but the relatively low decrease in viability, cell counting was used to monitor the proliferation of BC cell lines. Figure 6 shows the effect of BRCE treatment on the total number of viable cells after 12 h, 24 h, and 30 h. After 12 h, there were no significant differences in the number of viable cells between those which were not exposed to the treatment and those exposed to 100 μg/ml (*p* > 0.05, not significant; (MCF-7 *p* = 0.007; T47D *p* = 0.478; SKBR3* p* > 0.999; MDA-MB-231 *p* = 0.075; MDA-MB-468 *p* = 0.729) except BT-474 (*p* = 0.0103), whose cell population was smaller as the concentration of BRCE increased.

However, with prolonged exposure to the treatment (24–30 h), the population of viable cells was significantly reduced. After 30 h, the cell population exposed to 100 µg/ml was 57.9 (± 0.9)% (MCF-7) (*p* = 0.002), 29.4 (± 0.7)% (T-47D) (*p* < 0.0001), 11.7 (± 0.4)% (BT-474) (*p* < 0.0001), 64.6 (± 7.2)% (SK-BR-3) (*p* = 0.0009), 19.6 (± 0.9)% (MDA-MB-231) (*p* < 0.0001), 45.5 (± 1.6)% (MDA-MB-468) (*p* = 0.0002), of the control without treatment. Table 2 indicates the concentration required to reduce to half the population of viable cells.

**Table 2:** Half maximal inhibitory concentration (IC_50_) (µg/ml) of BRCE from *Hfx. mediterranei* R-4 on the viable cell concentration of representative BC cell lines for each intrinsic subtype at 30 h. ***p < 0.0005, ****p < 0.0001.

Cell line	Viable cells/ml IC_50_ (µg/ml)
184A1	> 100
MCF-7	78.6 (± 2.3)****
T-47D	52.7 (± 2.3)****
BT-474 50K	67.4 (± 1.6)****
BT-474 25K	42.4 (± 4.3)****
SK-BR-3	83.0 (± 4.2)***
MDA-MB-231	51.8 (± 0.5)****
MDA-MB-468	63.9 (± 2.7)****

The normal mammary cell line (184A1) was not significantly affected by the treatment regarding cell size, viability, adhesion, and concentration.

## Discussion

One of the best advantages of xCELLigence RTCA system technology is that it allows observing growth responses to treatments on cell lines in real-time without chemicals and dyes as in other conventional cytotoxicity analyses^34^. Apart from that, the innovative technology of xCELLigence was crucial for obtaining continuous measurements of cell adhesion rather than a single-time quantification.

This system measures the electrons that flow between the gold microelectrodes placed at the bottom of the microtiter plate using an electrically conductive solution, such as a cell culture medium. Once cells have adhered to the plate, the electron flow between electrodes and the conductive solution is blocked. The equipment translates this impedance into a unit called cell index that allows monitoring and quantifying cell proliferation and attachment^34^. Cell index depends on cell number, morphology, size, and attachment strength.

The most affected cell lines in terms of cell index were BT-474 (25,000 initial cell density), MCF-7, and T-47D. The sensitivity of the MCF-7 cell line to haloarchaeal carotenoids has been recently reported^20^. However, the effect on cell adhesion had not been addressed before this work. Luminal A and luminal B were more sensitive to the treatment than the rest of the subtypes. Luminal A is the most frequently diagnosed subtype, representing between 50 and 60% of cases^36^. Despite this, Luminal A tumours generally imply a good patient prognosis since strategies that block estrogen receptor function can be used^31,37^. Although these treatments are usually effective, over time, tumours evolve and might become resistant to these hormone-based drugs^38^. The fact that these carotenoids can act selectively on cancer cells is very promising. Luminal B usually behaves more aggressively than Luminal A tumours since they are associated with a higher probability of relapse and metastasis^30,39,40^.

The results obtained for triple-negative cell lines are also relevant, considering the aggressiveness that these tumours usually present, with a mortality rate of 40%^41^. The particularity of triple-negative tumours is that, given their phenotype, they do not respond to endocrine or molecular targeted therapy^42^. Consequently, they can only be treated with chemotherapy, resulting in poor outcomes. Therefore, developing or discovering new drugs or coadjuvants is necessary.

A selective effect is crucial for effective cancer treatment to exert high antitumor activity and minimal toxicity to normal tissues. An SI > 1.0 indicates that a compound has higher efficacy against tumour cells than against normal cells^35^. This study demonstrates that BRCE from *Haloferax mediterranei* exerts a selective effect in all tested breast cancer cell lines representative of the four different intrinsic subtypes. These results agree with the selective effect of haloarchaeal carotenoids. Previous research reported that *Natrialba* pigments present an SI > 2, higher than the SI for the chemotherapeutic drug 5-fluorouracil (SI < 1.7)^20^.

The observed decrease in cell index indicates a reduction in the detected adhesion of the cells to the plates. However, from these data is difficult to conclude if it is due to reduced cell proliferation, cell death, or a change in the morphology of the cells. For this reason, the diameter, viability, and total number of viable cells per ml were studied in parallel.

Although the advantages of using the xCELLigence technology are numerous, one limitation shall be considered in this field. Since it is a relatively new technique, there are fewer studies on natural compounds^43–47^ and none on carotenoids than those using more standardized methods, such as MTT^21,22,48–52^. On that account, this makes comparing results with previous research more difficult.

The analysis of viable cell diameter, cell viability, and the total number of viable cells was essential to determine the effect of *Hfx. mediterranei* BRCE on BC cells. Cell diameters were significantly reduced, and morphology alterations were detected in all tested BC cell lines exposed to the BRCE treatment. This effect was also reported for HepG2 cells testing extract from another haloarchaeon and morphological modifications^22^. In addition, the loss of the standard shape has been previously reported in the MCF-7 cell line in response to haloarchaeal carotenoids^20^.

One of our main objectives in this study was to determine if *Hfx. mediterranei* BRCE could affect BC cell viability, following the few studies previously published. Our results showed that BRCE treatment hindered cell viability, but it did not reduce it below 50% in most cases. MDA-MB-468 and MCF-7 were the most affected cell lines. The sensitivity of the MCF-7 cell line to haloarchaeal carotenoids has been recently reported with an IC_50_ value of 21 µg/ml^20^.

Many studies have explored the antiproliferative activities of carotenoids, but few are focused on haloarchaeal ones. Results reported by Hou and Cui coincide with the results here displayed since they described that carotenoid extracts from *Halogeometricum limi* (0.72 µg/ml) and *Haloplanus vescus* (0.045–0.72 µg/ml) exerted anticancer activity on HepG2 cells, leading to a decrease of 23% and 27% viability, respectively^21^. Recent research indicated that *Natrialba *sp. M6 pigments could reduce the viability of different cancer cell lines with concentrations ranging between 21.18 and 38.24 µg/ml^20^. The differences observed between studies might be related to the composition of the extract since it has been recently reported how the culture conditions and the species influence it^12^.

The sensitivity of the triple-negative cell line MDA-MB-468 should be highlighted. An earlier study has reported this responsiveness to lycopene due to G_0_/G_1_ cell cycle arrest apoptosis induction^53^, which was in agreement with a similar effect on MDA-MB-231^54^. This research on lycopene also demonstrated that it could inhibit protein kinase B (Akt) and mammalian target of rapamycin (mTOR) activation, although the mechanism remains unclear. Recent research has described the potentiating effect of fucoxanthin combined with doxorubicin, a chemotherapeutic drug^55^. Further research will be necessary to elucidate the mechanism of action behind the impact of haloarchaeal carotenoids on BC cell lines.

The normal mammary cell line 184A1 was not significantly affected by the treatment in terms of cell size, viability, adhesion, and concentration, which is in line with previous researchers reporting similar selective effects on cancer cell lines using *Natrialba *sp. M6 carotenoids^20^. In addition, lutein has been described to selectively target cancer cells by exerting a pro-oxidant effect since it increases ROS generation in triple-negative cells. In contrast, normal cells remain unaffected^56^.

Regarding Luminal A cell lines (MCF-7 and T-47D), cell adhesion, viability, and viable cell concentration were altered significantly in both cell lines, whereas cell diameter was differentially influenced. The Luminal subtype seems to be sensitive to haloarchaeal carotenoid treatment^.^

Similarly, the Luminal B cell line (BT-474) was one of the most affected cell lines since the cell index values differed clearly from the control, probably due to a delay in growth since viability was not strongly influenced, whereas the cell concentration was. However, BT-474 belongs to those Luminal B tumours with overexpression of HER2 (ER^+^ PR^−^ HER2^+^). Therefore, more studies would be needed to explore the effects of this treatment also on cell lines representing Luminal B without HER2 overexpression (ER^+^ PR^−^ HER2^−^).

HER2^+^-enriched cell line SK-BR-3 responded differently to Luminal A representatives. In this case, SK-BR-3 is one of the least responsive cell lines in terms of cell concentration, but as opposed to this, the cell size was strongly altered.

Finally, the results of triple-negative representatives (MDA-MB-231 and MDA-MB-468) are encouraging. Cell adhesion was significantly impaired due to the several variables studied in this article. The diameter, viability, and cell concentration were significantly diminished in both cell lines at the highest concentrations. MDA-MB-468 was more sensitive to treatment than MDA-MB-231, which makes sense considering that MDA-MB-231 is a Basal B cell line with features of claudin-low triple-negative tumours, which usually behave more aggressively than Basal A triple-negative tumours, from which MDA-MB-468 cell line is representative^33,57^. In addition, MDA-MB-231 has higher mutant Tp53 expression than MDA-MB-468^33^. MDA-MB-231 also has one of the highest described invasive potentials for BC cell lines^58^.

CD24^−/low^/CD44^+^, cell surface markers, have been described for this cell line, implying stem cell properties^59^. However, despite this, both cell lines responded favourably, opening the door for further studies in this area.

The molecular mechanisms involved in the effect of haloarchaeal carotenoids in BC cell lines are not precise yet. However, some researchers have described that carotenoids from halophilic archaea might exert an apoptotic influence via caspase activation. They can also inhibit MMP-9 protease, which is involved in invasion, angiogenesis, and metastasis in cancer^20^. In addition, lycopene suppresses cell proliferation, possibly through p53 and Bax mRNA upregulation^60^, and can modulate the gap junction intercellular communication in BC^61^.

It is also worth mentioning that recent research has discussed how *Hfx. mediterranei* cell culture conditions might influence carotenoid extracts composition, thus, altering their antioxidant, antiglycemic, and antilipidemic properties^12^. This study explores the antiproliferative activity of a *Hfx. mediterranei* carotenoid extract obtained from a cell culture under certain conditions (12.5% SW (w/v), 1.5% glucose, 36.5 °C). For that reason, further studies are required to determine if variations in the composition of BRCE due to culture conditions also affect the antiproliferative effect. Currently, very little information is available about the pharmacological applications that haloarchaeal carotenoids might have. This study provides for the first time the effect of BRCE on different BC cell lines, representative of the four main types of BC tumours. This work presents new insights into the biological activity and potential biomedical applications of complex haloarchaeal carotenoids. The experiments presented here confirmed that *Haloferax mediterranei* BRCE was cytotoxic to all tested BC cell lines representative of each BC subtype.

In contrast, normal mammary tissue cells were not sensitive to the treatment. One of the main limitations of this work is that the contribution of the individual carotenoids conforming the extract to its antiproliferative activity is yet unknown. Consequently, more experimental evidence is required to answer the questions about these natural compounds' potential health benefits and mechanism of action.

## Conclusion

In conclusion, we have demonstrated that BRCE exerts selective antiproliferative and cytotoxic effects on BC cell lines representative of each intrinsic subtype. Notably, its effect on triple-negative cell line viability is promising. Therefore, considering the aggressiveness and the lack of targeted treatments for this intrinsic subtype, BRCE may be postulated as a potential therapeutic strategy. Finally, our study provides insights into potential applications of haloarchaeal carotenoids and enables advances in understanding its effects in vitro.

## Data Availability

The datasets analysed during the current study are available from the corresponding author upon reasonable request.
